# Decoding the Phosphatase Code: Regulation of Cell Proliferation by Calcineurin

**DOI:** 10.3390/ijms23031122

**Published:** 2022-01-20

**Authors:** Takahiro Masaki, Midori Shimada

**Affiliations:** Department of Biochemistry, Joint Faculty of Veterinary Science, Yamaguchi University, 1677-1 Yoshida, Yamaguchi 753-8511, Japan; g029tb@yamaguchi-u.ac.jp

**Keywords:** cancer, cell cycle, intracellular calcium ions, calcineurin, dephosphorylation

## Abstract

Calcineurin, a calcium-dependent serine/threonine phosphatase, integrates the alterations in intracellular calcium levels into downstream signaling pathways by regulating the phosphorylation states of several targets. Intracellular Ca^2+^ is essential for normal cellular physiology and cell cycle progression at certain critical stages of the cell cycle. Recently, it was reported that calcineurin is activated in a variety of cancers. Given that abnormalities in calcineurin signaling can lead to malignant growth and cancer, the calcineurin signaling pathway could be a potential target for cancer treatment. For example, NFAT, a typical substrate of calcineurin, activates the genes that promote cell proliferation. Furthermore, cyclin D1 and estrogen receptors are dephosphorylated and stabilized by calcineurin, leading to cell proliferation. In this review, we focus on the cell proliferative functions and regulatory mechanisms of calcineurin and summarize the various substrates of calcineurin. We also describe recent advances regarding dysregulation of the calcineurin activity in cancer cells. We hope that this review will provide new insights into the potential role of calcineurin in cancer development.

## 1. Introduction

Intracellular calcium ions (Ca^2+^) act as pleiotropic secondary messengers in key signaling pathways involving a variety of cellular functions. While the extracellular Ca^2+^ concentration is 11.5 mM, the steady-state intracellular Ca^2+^ concentration is kept very low at several tens of nanomoles. It is well known that the concentration of Ca^2+^ varies greatly in different cellular compartments, for example, intracellular calcium, 100 nM; endoplasmic reticulum (ER), 0.5–1 mM; and mitochondria, 100–200 nM [1].

The binding of a hormone or growth factor to a G protein coupled receptor (GPR) or a tyrosine kinase receptor (RTK) initiates the Ca^2+^ signaling cascade. The activation of such receptors transmits the signals to phospholipase C (PLC), which cleaves phosphatidylinositol 4,5-biphosphate (PIP_2_) to produce diacylglycerol (DAG) and inositol-1,4,5-trisphosphate (InsP_3_). InsP_3_ then binds to the InsP_3_ receptor (InsP_3_R) and stimulates the release of Ca^2+^ from intracellular stores, such as ER [2], and allows for the entry of Ca^2+^ [3,4] from the extracellular space. Spatially and temporally coordinated Ca^2+^ release via InsP_3_R is regulated by complex feedback mechanisms [1].

Changes in Ca^2+^ concentration trigger signal transduction, which regulates a wide range of cellular events such as gene expression, cell cycle, cell motility, autophagy, and apoptosis [1,5]. Local changes in intracellular Ca^2+^ diffuse through the cell and have effects on the distal sites [6]. Long-term intracellular Ca^2+^ increases in the mitochondria and causes the release of cytochrome c and subsequently triggers apoptosis [7]. Due to the diverse roles of Ca^2+^, the dysregulation of calcium homeostasis can impair cell function and trigger cell death, and by doing so, may contribute to heart disease, cancer, and mental disorders. As a result, Ca^2+^ signals must be tightly regulated during the cell cycle. Indeed, previous studies have characterized Ca^2+^ transients during the cell cycle [8,9]. For example, Pande et al. (1996) measured the intracellular levels of free cytoplasmic Ca^2+^ in rat fibroblasts and found that complex changes occurred in calcium levels during the cell cycle [10]. They showed that the levels of Ca^2+^ were the lowest at the beginning of the G_1_ phase but were subsequently increased at the G_1_/S border.

Ca^2+^ oscillations are narrow in the sense of periodic rise and fall, while Ca^2+^ concentration alterations are broad in the sense of simple Ca^2+^ concentration changes. To facilitate different cellular processes, intracellular downstream effectors decode the frequency, amplitude, and duration of intracellular Ca^2+^ oscillations [11]. The most widely known family of calcium downstream effectors is calmodulin (CaM). CaM is highly conserved among eukaryotes with four EF-hand Ca^2+^ binding sites. CaM is involved in the regulation of cell proliferation and cell cycle through CaM-dependent phosphorylation/dephosphorylation events [12]. When Ca^2+^ binds to CaM, the Ca^2+^/CaM complex can interact with and control target proteins such as serine/threonine phosphatase calcineurin (CaN) and the family of Ca^2+^/CaM-dependent protein kinases (CaMK).

The molecular mechanisms through which Ca^2+^/CaM regulates cell cycle progression, especially through the activation state of the cyclin-dependent kinase (CDK) complex, is of great interest, and therefore has been studied intensively. It has been shown that although all eukaryotic cells require Ca^2+^ signaling for cell proliferation, some transformed cells and tumor cell lines are less dependent on Ca^2+^ for cell growth [13,14]. Ca^2+^-mediated signaling pathways, therefore, play important roles in tumorigenesis, tumor progression, metastasis, and invasion [6]. In this review, we discuss the latest findings of CaN in cell proliferation and its potential as a new therapeutic target in the treatment of cancer.

## 2. Cell Cycle and CDK

Cell division is precisely regulated by a series of CDKs, whose activity is dependent on the binding to different cell cycle-specific cyclins [15]. Cyclins confer substrate specificity and form CDK/cyclin complexes, which activate or inactivate target proteins by phosphorylation, and orchestrate coordinated cell cycle progression. In addition to CDK association with cyclins, the activity of CDKs is tightly regulated by several mechanisms, including phosphorylation, binding of CDK inhibitors (CKIs), and subcellular localization of CDK/cyclin complexes [16].

One of the critical targets of the CDK-cyclin complex in the G_1_ phase is the retinoblastoma protein (Rb). In the early G_1_ phase, CDK4/6-cyclin D initiates the phosphorylation of Rb, and, subsequently, CDK2-cyclin E phosphorylates Rb [17,18,19,20]. Phosphorylation of Rb releases the E2F transcription factor, and promotes the expression of the genes required for cell cycle progression [21,22]. This enables cells to pass through the restriction point at the G_1_/S boundary and to commence the S phase. CDK2-cyclin A plays an important role in S phase progression through the phosphorylation of proteins involved in DNA replication [23,24]. As cells enter the S phase, the CDK2-cyclin A complex is activated and remains activated throughout the G_2_ phase [25,26]. In the late G_2_ phase, CDK1-cyclin B is activated, allowing for the entry of cells into mitosis [27]. During G_2_/M transition, CDK1-cyclin A activity is necessary for prophase initiation [28].

## 3. Calcium, Calmodulin, and Calmodulin-Dependent Protein Kinases in Cell Proliferation

In mammalian cells, Ca^2+^ is required at several different points during the cell cycle. Cells are most sensitive to the depletion of extracellular Ca^2+^ at early G_1_ and near the G_1_/S boundary [12,29]. In early G_1_, Ca^2+^ regulates the expression of immediate-early genes, such as *FOS*, *JUN*, and *MYC*. Ca^2+^ is also required for Rb phosphorylation at the G_1_/S boundary. Spontaneous Ca^2+^ oscillations at the G_1_/S phase transition have been described in synchronized immortalized cell lines. These Ca^2+^ oscillations are accompanied by DNA replication [30].

Once hormone receptors are activated, intracellular Ca^2+^ levels increase. Ca^2+^ regulates certain targets directly and others indirectly through Ca^2+^-binding proteins, such as CaM. Ca^2+^/CaM activates various pathways involved in the regulation of cellular processes, such as secretion, cell motility, ion homeostasis, and gene transcription. CaM also modulates a large number of intracellular enzymes, including protein kinases, protein phosphatases, phosphodiesterases, adenylyl cyclases, and ion channels [31].

**Figure 1:**
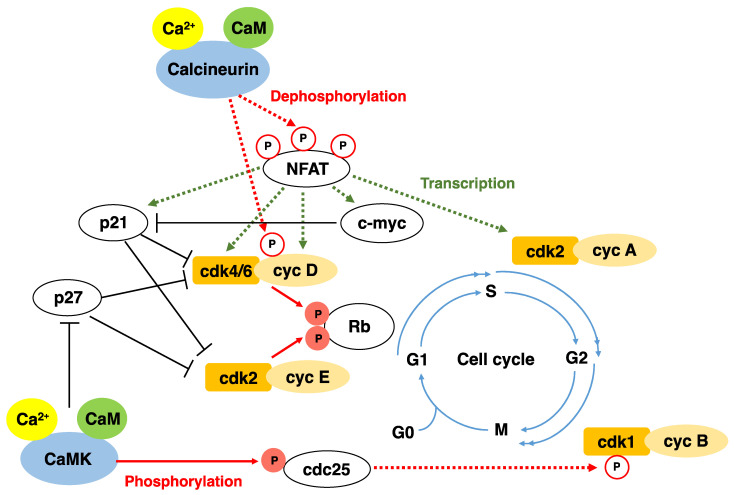
Regulation of cell cycle progression by calcineurin and CaMK. CaN dephosphorylates the NFAT transcription factor, which in turn activates p21, cyclin D1, CDK4, c-myc, and cyclin A. CaN also stabilizes cyclin D1 by dephosphorylation. Furthermore, the CaN/NFAT pathway and its downstream target c-myc regulate p21. p21 is a well-known inhibitor of CDK2-cyclin E and CDK4/6-cyclin D. CaMK negatively regulates the expression of p27, which is an inhibitor of CDK4-cyclin D and CDK2-cyclin E. CDK2-cyclin E and CDK4/6-cyclin D complexes phosphorylate Rb, leading to the activation of E2F1 and the subsequent G1/S progression. In G2/M, CaMK phosphorylates and activates cdc25, leading to downstream dephosphorylation and the activation of CDK1. Solid red lines indicate phosphorylation, red dotted lines indicate dephosphorylation, and green dotted lines indicate transcriptional activation.

CaM is required for cell cycle progression through G_1_, specifically the G_1_/S transition. Consistent with this notion, CaM antagonists block cell cycle progression in early or late G_1_ phases [32]. Furthermore, the exit from mitosis is sensitive to changes in the concentration of CaM [33]. Upon binding with Ca^2+^, CaM undergoes a major conformational change, and forms a Ca^2+^/CaM complex to interact with a variety of target proteins such as cellular kinases [34]. The best-studied kinases involved in Ca^2+^/CaM signaling are the CaMKs [35,36]. CaN and CaMK play important roles in cell cycle progression by activating or inhibiting key cell cycle regulators (Figure 1). The CaN/NFAT pathway contributes to inducing the transcription of cyclin D1 [37] and CDK4, and stabilizes cyclin D1 by dephosphorylating Thr286 of cyclin D [38]. The same pathway also regulates p21, which inhibits CDK2-cyclin E and CDK4/6-cyclin D. In colorectal cancer cells, NFATc1 represses p21 expression by binding to the c-myc promoter [39]. In contrast, in breast cancer cells, the CaN/NFAT pathway promotes p21 transcription [40]. In addition, the transcription of cyclin A, which regulates the progression of S and G_2_ phases, is also activated by the CaN/NFAT pathway [41]. CaMK regulates cell cycle progression from G_1_ to S phase [42,43], and negatively regulates the expression of p27, which is an inhibitor of CDK4-cyclin D and CDK2-cyclin E [44]. In the G2/M phase, CaMK phosphorylates and activates CDC25, leading to the dephosphorylation and activation of CDK1 [45]. As a result, the inhibition of CaMK causes cell cycle arrest at G_1_/S [46,47,48] or G_2_/M transition [49], with decreased or increased expression levels of cyclin D1 and p27, respectively. G1 arrest by CaMK inhibition also accompanies CDK4 and CDK2 inhibition [43,50,51]. Not surprisingly, CaMK appears to be highly active in tumor cells, such as myeloid leukemia, glioma, and endometrial and thyroid carcinoma, where it contributes to accelerated cell proliferation [46,52,53].

## 4. Characteristics of Calcineurin

CaN, also known as protein phosphatase 2 B (PP2B), is a serine/threonine protein phosphatase that is conserved in all eukaryotes [54,55,56,57]. CaN is a heterodimer composed of a catalytic subunit, calcineurin A (CnA), and a Ca^2+^-binding regulatory subunit, calcineurin B (CnB). In mammals, three independent genes, *PPP3CA*, *PPP3CB*, and *PPP3CC*, encode CnAα, CnAβ, and CnAγ, respectively. CnAα and CnAβ exhibit ubiquitous expression patterns, whereas the CnAγ expression is restricted to the testis and brain [58,59,60,61]. The CaN regulatory subunits CnB1 and CnB2 are encoded by two genes (*PPP3R1* and *PPP3R2*, respectively). The CnB1 protein is expressed ubiquitously, whereas the CnB2 protein is specifically expressed in the testes. CnA contains an amino-terminal catalytic domain followed by a CnB-binding domain, a CaM-binding domain, and an autoinhibitory domain. CaN also contains a nuclear localization signal (NLS) in the catalytic domain and a nuclear export signal (NES) in the carboxyl terminus. The autoinhibitory domain of CnA blocks the catalytic site and the NLS [56,62]. CnB contains four EF-hand Ca^2+^-binding motifs and an amino-terminal myristylation site. CaN is activated by the increased intracellular Ca^2+^ concentration in the cell and plays essential roles in multiple signaling processes [63]. The binding of Ca^2+^/CaM to the regulatory domain leads to the attenuation of autoinhibition, followed by dramatic enzymatic activation. Of note, although there are multiple kinases that are regulated by CaM, and CaN is the only phosphatase directly regulated by Ca^2+^/CaM.

As mentioned above, CaN is widely distributed in various mammalian tissues and is particularly abundant in neural tissues [64]. However, the abundance and sub-cellular localization of CnAα and CnAβ are different. CnAα is more abundantly expressed than CnAβ; furthermore, CnAα localizes in the nucleus while CnAβ localizes in the cytoplasm [65]. Interestingly, although CaN localizes predominantly in the cytoplasm of unstimulated cells [61,66,67] in response to elevated Ca^2+^ concentrations, and a small portion of CaN can translocate to the nucleus and interact with the target substrates [68].

## 5. Mechanisms Regulating Calcineurin Activity

Several proteins have been reported to inhibit CaN, including AKAP79 (A-kinase anchoring protein-79) [69], PMCA2 (plasma membrane calcium ATPase 2) [70], CHP (calcineurin homologous protein) [71,72], Cabin/Cain [73,74], calcipressin/RCAN/DSCR/CSP [75,76,77], and FK506-binding protein (FKBP) 38 [78]. Subcellular localization of CaN is regulated by its interaction with AKAP79, a scaffold protein that anchors CaN at distinct subcellular locations, leading to the inhibition of CaN [67,69,79]. In breast cancer cells, PMCA2 interacts with and sequesters CaN in the membrane, and suppresses the activation of the CaN-NFAT pathway [70].

CHP competes with CnB to bind to CnA, and inhibits CaN activity [71,72]. While Cabin1/cain inhibits CaN by interacting with CaN in a phosphorylation-dependent manner through a binding site on CaN, which is distinct from that of the drug–immunophilin complex [73,74]. Calcipressin/RCAN/DSCR/CSP has also been identified as a CnA-binding protein that inhibits CaN activity [77,80]. A conserved peptide (FLISPPxSPP) of the calcipressin family is phosphorylated and functions as a binding site for CaN. As the expression of calcipressin is induced by CaN, it functions as a feedback inhibitor of CaN signaling. Calcipressin/RCAN/DSCR/CSP binds CaN at the same site as NFAT and other substrates, with competition for binding between these molecules being a possible regulatory mechanism [81]. FKBP38 targets BCL-2 to the mitochondria and inhibits apoptosis. The same protein also binds to and inhibits CaN, even in the absence of FK506 [78]. Furthermore, histone H1 inhibits CaMKII and CaN by blocking CaM autophsophorylation [82]. CaN is also inactivated by the oxidation of key methionine residues [83,84,85,86]. Conversely, CaN is activated by the intramolecular cleavage by two different proteases, caspase 3 and Ca^2+^-dependent protease calpain [87,88,89]. Several studies have identified that CaN activity is regulated by its phosphorylation. The CaMKII-mediated phosphorylation of CaN on Ser197 inhibits the CaN activity [90,91,92]. This phosphorylation is blocked by Ca^2+^/CaM binding to CaN. In contrast, although the auto-dephosphorylation of CaN is very slow, it can be rapidly dephosphorylated by protein phosphatase IIA [91].

## 6. Functions of Calcineurin/NFAT

Cyclosporine A and FK506 are well-characterized immunosuppressive agents that prevent organ transplant rejection [93,94,95]. These compounds bind tightly to endogenous cytoplasmic cyclophilin A or FKBP12, respectively. Interestingly, cyclophilin A or FKBP12, in complex with cyclosporine A or FK506, bind to CaN and block the access of the CaN substrate to the active site of the CaN [96]. This indicates the possibility that the immunosuppressive effects of these drugs could be, in part, caused by the disruption of CaN functions. Consistent with this view, it has been show that CaN inhibition by cyclosporine A or FK506 delays G_1_/S progression in various cell types [97,98,99,100]. Mechanistically, cyclosporin A induces the expression of the cyclin inhibitor p21 and a reciprocal reduction in cyclins A and E, leading to CDK2 inactivation [101,102].

The most studied substrates of CaN are the family of nuclear factors of activated T-cell (NFATc or NFAT) transcription factors [57,103]. Four of the five members of the NFAT protein family, NFATc1 (NFAT2), NFATc2 (NFAT1), NFATc3 (NFAT4), and NFATc4 (NFAT3), are regulated by calcium signaling. In T cells, dephosphorylation of NFAT by CaN changes the structure of the NFAT protein, exposing the NLS, which is then relocated to the nucleus to regulate the transcription of immune function-associated genes [104,105,106]. In addition to T cells, NFATs are also present in a wide variety of cells and tissues, and dephosphorylation of NFAT by CaN activates transcription in the neurons and astrocytes [107,108], and also in the heart and skeletal muscle [109,110,111,112]. Of note, NFAT has been shown to regulate cell cycle-related genes and promote cell cycle progression [103,113,114,115]. Furthermore, both CaN and NFATc1 regulate cyclin D1 transcription [37]. In addition, Camp-responsive element binding protein 1 (CREB1) transcription factor, which binds to the cyclin D1 promoter and induces cyclin D1 mRNA expression, is also regulated by CaN [98]. The other key target of NFATc1 in cell cycle progression is c-Myc. CaN-mediated dephosphorylation of NFATc1 activates *MYC* transcription, allowing the cell to proceed to the S phase [116,117]. NFATc1 binds directly to the NFAT site in the *MYC* promoter [115]. In addition, NFATc1 increases c-myc transcription by activating the ERK1/2/p38/MAPK signaling pathway [118] or inducing histone acetylation, resulting in the binding of ELK1 to the *MYC* promoter [119]. Thus, NFATc1 upregulates *MYC* transcription via multiple mechanisms.

Given the diverse expression and function of each NFAT isoform, its dysregulation is known to be associated with tumorigenesis, Alzheimer’s disease, and the development of autoimmune and inflammatory diseases. A novel therapeutic strategy for treating NFAT-related diseases is to develop new ways to selectively regulate specific NFAT isoforms [120].

## 7. Other Substrates of Calcineurin

Approximately 600 proteins with conserved sequences that bind to CaN have been identified [121]. Many of the known CaN substrates possess PxIxIT and/or LxVP motifs [56]. Such substrates include transcription factors, proteins involved in cell cycle and apoptosis, cytoskeletal proteins, scaffold proteins, membrane channels, and receptors (Table 1) [56,58,122,123].

Key proteins associated with tumor development such as myocyte enhancer factor 2 (MEF2), kinase suppressor of ras 2 (KSR2), DAXX, c-Jun, and nuclear factor I (NFI), are known CaN substrates [128,129,134,136,138]. Furthermore, CAN dephosphorylatesd the pro-apoptotic Bcl-2 family member BAD [126] and the apoptosis promoting factor ASK1 [125]. Dephosphorylation of another apoptosis related factor CaMKIIγ is promoted by CaN, leading to the nuclear translocation of CaMKIIγ. Nuclear translocation of TFEB, a master transcriptional regulator of lysosome biogenesis and autophagy, also requires dephosphorylated by CaN [127,145]. In addition, while DARPP-32 and ElK-1 are inactivated by CaN, other targets such as nitric oxide synthase, NHE1, and TRESK are activated [142,146,147,148,149]. CaN is activated through cytoplasmic Ca^2+^ increase caused by mitochondrial depolarization. Activated CaN dephosphorylates DRP1, which then relocates to the mitochondria and promotes mitochondrial fission [130]. In neurons, CaN dephosphorylates dynamin1. This promotes the endocytosis of TRKA receptors and subsequent axonal growth [131]. CaN-dependent dephosphorylation of GluA1, a component of AMPA receptors (AMPARs) at the synapses, leads to the removal of AMPARs from the synapse and endocytosis [133], while dephosphorylation of Myosin phosphatase target subunit 1 (MYPT1, a component of MP) by CaN causes MP to acquire phosphatase activity [137].

**Figure 2:**
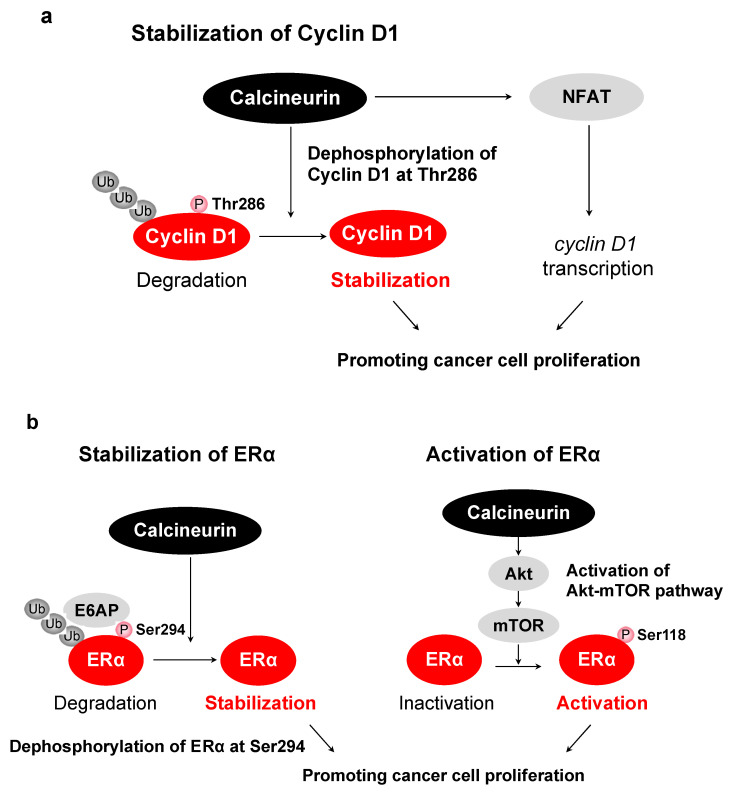
A new role of calcineurin in cell proliferation. (**a**) CaN regulates the stability and transcription of Cyclin D1. Phosphorylation of Thr286 of cyclin D1 promotes polyubiquitination and the subsequent degradation of cyclin D1. CaN stabilizes cyclin D1 by dephosphorylating the Thr286 of cyclin D1. Furthermore, NFAT activated by CaN induces the transcription of cyclin D1. Thus, CaN contributes to cancer cell proliferation by regulating the stability and transcription of cyclin D1. (**b**) Calcineurin regulates the stability and activity of Erα. Phosphorylation of Ser294 of ERα promotes polyubiquitination by the E3 ligase E6AP and the subsequent degradation of ERα. By dephosphorylating the Ser294 of ERα, CaN releases E6AP from ERα and stabilizes ERα (left). Furthermore, CaN activates the Akt-mTOR pathway via the phosphorylation of Ser118 of ERα (right). Thus, CaN contributes to the proliferation of cancer cells by regulating the stability and activity of ERα.

Recently, we found that CaN inhibits cyclin D1 degradation by dephosphorylation of the Thr286 residue [38] (Figure 2a). Treatment with CN585, which is a specific inhibitor of CaN phosphatase activity, or by the immunosuppressant FK506, inhibits breast cancer cell proliferation accompanied by delayed G_1_/S progression mediated by the degradation of cyclin D1. FK506 also decreases the protein level of CDK4 via a yet unknown mechanism. Taken together, CaN activates CDK4-cyclin D1 through multiple mechanisms to promote cell cycle progression [12,38]. The overexpression of cyclin D1 has been linked to the development and progression of cancer [150]. It is also known that increased levels of cyclin D1 could frequently result from deregulated proteasomal degradation [151]. This indicates that the selective inhibition of CaN could be an effective method for the treatment of cancers that are characterized by cyclin D1 overexpression. We also found that the stability and activity of ERα, a key molecule for estrogen-dependent cancer progression, is mediated by CaN [132] (Figure 2b). CaN dephosphorylates ERα on Ser294 and activates the mechanistic target of rapamycin (mTOR). CaN is also dephosphorylate MAP2, RIIα, RCAN1, and Tau, but the significance of these dephosphorylations remains unclear [124,135,143,144].

Intriguingly, CaN may possess roles that are independent of its dephosphorylation activity. CnAβ1 functionally activates the PDK1-Akt phosphorylation cascade, increasing the phosphorylation of its target, Ser253 of FOXO3a, and the inhibition of its nuclear translocation. The inhibitory effect of FOXO3a by CnAβ1 regulates myoblast proliferation and prevents myotube atrophy under starvation conditions. Of note, this function of CnAβ1 does not require a phosphatase activity, suggesting that CaN functions independently of its dephosphorylation activity [152].

## 8. Activation of Calcineurin/NFAT Pathway in Cancer

The CaN/NFAT pathway is activated in multiple cancers, including breast cancer [153], lung cancer [154], prostate cancer [155], ovarian cancer [118], pancreatic cancer [115], liver cancer [156], colorectal cancer [39], glioblastoma [138,157], melanoma [158], leukemia [159,160], and lymphoma [161,162]. The dysregulated CaN/NFAT pathway observed in cancer is summarized in Table 2.

The mechanisms that drive CaN activation in cancer are not well understood. In some cancers, CaN hyperactivation has been associated with mutations. For example, the EL4 murine T-cell lymphoma cell line expresses a mutant form of CaN, in which the aspartic acid at position 477 is mutated to asparagine and the negative regulation of the phosphatase activity by the autoinhibitory domain is impaired by this mutation [167]. In contrast, the SML B-cell lymphoma cell line expresses a truncated version of CnA, which results in constitutive activation of CaN [168]. It has been reported that the expression of Ca^2+^ channels that regulate the intracellular Ca^2+^ concentration is involved in the activation of CaN. TRPv6 (TRP vanilloid family member 6), a Ca^2+^-selective ion channel, is highly expressed in prostate cancer and is a prognostic marker [169,170]. Increased expression of TRPv6 induces more Ca^2+^ to enter the cell, which in turn promotes NFAT activation [171]. It is also possible that the inflammatory response activates CaN [172,173,174]. Consistent with this notion, in intestinal cancer, changes in the bacterial community activate Toll-like receptor (TLR) signaling, which leads to subsequent calcium entry and CaN activation [175]. Moreover, inflammatory responses are activated in many cancers, and it is well understood that chronic inflammation is a risk factor for tumorigenesis [176]. Recently, we found that a high expression of CaN was correlated with a poor prognosis regarding the outcome of endocrine therapy in patients with ERα-positive breast cancer [132], which indicates that the selective inhibition of CaN could be effective to treat such cancers.

Highly activated CaN is supposed to activate its substrates, including NFAT, to promote proliferation. Similar to CaN, its target NFAT is also constitutively activated or overexpressed in numerous cancers and may contribute to cancer development and progression [177]. Conversely, the expression of NFATc1 is suppressed in several types of cancer, and a reduction of NFATc1 has been shown to be linked with aggressiveness and malignancy of cancer [164,165,166].

## 9. A Therapeutic Perspective for Cancer

Owing to the high frequency of CaN/NFAT activation in cancer and the contribution of these molecules in cancer progression, the CaN/NFAT pathway could be a potential therapeutic target. Indeed, the anticancer effects of CaN inhibitors have been studied extensively in the past. For instance, cyclosporine A or FK506 induced apoptosis and rapid tumor clearance, resulting in the regression of leukemia [160]. Cyclosporine A or FK506 also inhibits tumor growth in the bladder and prostate xenografts in vivo [174,178,179]. In addition, cyclosporine A itself is also directly involved in tumor growth, as it increases TGFβ production [180], activates Ras [181], suppresses PTEN expression, and increases AKT activation [182].

There is growing acceptance that targeting dysregulated Ca^2+^ channels/transporters/pumps can provide promising potential for the treatment of cancer patients. [183]. Buffering nuclear Ca^2+^ concentrations alters the expression levels of the genes involved in cell proliferation, resulting in an antitumor effect [184]. Moreover, buffering nuclear Ca^2+^ reduces the growth rate of tumor cells without affecting the cells in normal tissues [185]. Further research is needed to clarify the differences in the sensitivity of normal and cancer cell growth to changes in nuclear Ca^2+^ levels.

## 10. Conclusions

Nuclear Ca^2+^ is involved in tumor growth and alters the expression of the genes involved in cell proliferation. Furthermore, previous studies have suggested that the modulation of nuclear Ca^2+^ signaling may be effective in cancer therapy. Activation of CaN and its downstream dephosphorylation has been identified as a mechanism by which nuclear Ca^2+^ regulates cell proliferation and cell cycle progression. As discussed above, dephosphorylation of proteins by CaN plays an important role in tumor formation and progression. Therefore, in the future, it will be necessary to identify the molecular mechanism of CaN activation and the substrates that promote cancer cell growth. Targeting the interactions of activated CaN with specific substrates in cancer cells, without affecting the normal immune function of CaN, may effectively inhibit the growth of cancer cells, leading to the establishment of new tumor-specific therapies.

## Figures and Tables

**Table 1:** Calcineurin substrates.

Calcineurin Substrates	Dephosphorylation Sites	Reaction by Dephosphorylation	Reference
12E8	Ser262, Ser356	ND	[124]
ASK1	Ser967	Promotes the dissociation of ASK1 from the 14-3-3 protein, resulting in the activation of ASK1	[125]
AT270	Thr181	ND	[124]
BAD	Ser155	Promotes heterodimerization of BAD and Bcl-xL, which induces apoptosis	[126]
CaMKIIγ	Ser334	Translocates CaMKIIγ to the nucleus	[127]
c-Jun	Ser243	Stabilizes c-Jun, promotes the interaction between c-Jun and Sp1	[128]
Cyclin D1	Thr286	Stabilizes cyclin D1, inducing G1/S progression	[38]
DAXX	Ser669	Promotes H3.3 uptake by DAXX	[129]
DRP1	Ser637	Splites the organelle by Drp1	[130]
Dynamin 1	Ser774, Ser778	Promotes endocytosis of TrkA receptors and axonal growth	[131]
ERα	Ser294	Stabilizes ERα and promotes the activity of ERα	[132]
GluA1	Ser845	Promotes removal of AMPARs from synapses and endocytosis	[133]
KSR2	Ser198, Thr287, Ser310	Activates ERK and induces membrane localization of KSR2	[134]
MAP2		ND	[135]
MEF2A	Ser221, Ser255, Ser408	Activates MEF2A and promotes the change from sumoylation to acetylation of Leu403	[136]
MYPT1	Thr696	Affects actin polymerization by activating MP and improves endothelial barrier function	[137]
NF1	ND	Activates transcription	[138]
NFATC1	Ser172	Promotes nuclear transfer of NFATC1	[139]
NFATC2 *	Five residues among following sites, Ser170, Ser173, Ser174, Ser176, Ser177, Ser179, Ser182, in SRR-1 domain	Promotes nuclear transfer of NFATC2	[140]
	Ser215, Ser219, Ser223 in SP-2 domain		
	Ser270, Ser276, Ser278, Ser282 in SP-3 domain		
	Ser328 in KTS motif		
NFATC4	Ser170	Promotes nuclear transfer of NFATC4	[141]
NHE1	Thr779	Inhibits NHE1 activity	[142]
PHF1	Ser396, Ser404	ND	[124]
RIIα	Ser95	ND	[143]
RCAN1	Ser108, Ser112, Thr124, Thr192	ND	[144]
Tau1	Ser199, Ser202	ND	[124]
TFEB	Ser142, Ser211	Promotes nuclear transfer of TFEB	[145]
TRESK	Ser276	Increases K+ current, decreases channel responsiveness to calcium signals	[146]

The amino acid residues of the protein indicated by asterisks (*) are dephosphorylated when the cells are stimulated with ionomycin. It is not known whether calcineurin directly dephosphorylates them. ND means not determined.

**Table 2:** Dysregulation of the calcineurin/NFAT pathway in cancer.

Factor	Alterations in Cancer	Types of Cancer	Reference
Calcineurin (CnA)	Overexpression	glioma (malignant gliomas, including grades III and IV astrocytomas)	[138]
		breast cancer (ER-α–positive)	[132]
	Activation	lymphomas (lymphoid malignancies)	[160]
	Overexpression, activation	colon cancer	[39]
NFATc1	Overexpression	ovarian cancer (clear-cell carcinoma)	[118,163]
		liver cancer (hepatocellular carcinoma)	[156]
		prostate cancer	[155]
		lymphomas (large B-cell lymphoma)	[162]
	Nuclear localization	lymphomas (diffuse large B-cell lymphomas)	[161]
		breast cancer (triple-negative)	[153]
	Suppression	liver cancer (hepatocellular carcinoma)	[164,165]
		lymphomas (anaplastic large cell lymphomas and classical Hodgkin’s lymphomas)	[166]
	Overexpression, nuclear localization	pancreatic cancer (pancreatic adenocarcinoma)	[115]
NFATc2	Overexpression	melanoma	[158]
		glioma (glioblastoma)	[157]
	Nuclear localization	lung cancer	[154]
NFATc1, NFATc3	Dephosphorylation	leukemia (T-cell acute lymphoblastic leukemia)	[159]
